# Frailty Increases the Risk and Frequency of Lower Respiratory Tract Infections in Older Adults: A Multicenter Prospective Cohort Study

**DOI:** 10.1093/ofid/ofag223

**Published:** 2026-04-24

**Authors:** Qing Zhang, Xiaoying Gu, Kang Chang, Yawen Ni, Chun Wang, Jie Zi, Baocheng Zhao, Xiaohui Zou, Bin Cao

**Affiliations:** Department of Pulmonary and Critical Care Medicine, China-Japan Friendship Hospital, Chinese Academy of Medical Sciences and Peking Union Medical College, Beijing, China; National Center for Respiratory Medicine; State Key Laboratory of Respiratory Health and Multimorbidity; National Clinical Research Center for Respiratory Diseases; Institute of Respiratory Medicine, Chinese Academy of Medical Sciences; Beijing Key Laboratory of Surveillance, Early Warning and Pathogen Research on Emerging Infectious Diseases; Laboratory of Clinical Microbiology and Infectious Diseases, Department of Pulmonary and Critical Care Medicine, Center of Respiratory Medicine, China-Japan Friendship Hospital, Beijing, China; National Center for Respiratory Medicine; State Key Laboratory of Respiratory Health and Multimorbidity; National Clinical Research Center for Respiratory Diseases; Institute of Respiratory Medicine, Chinese Academy of Medical Sciences; Beijing Key Laboratory of Surveillance, Early Warning and Pathogen Research on Emerging Infectious Diseases; Laboratory of Clinical Microbiology and Infectious Diseases, Department of Pulmonary and Critical Care Medicine, Center of Respiratory Medicine, China-Japan Friendship Hospital, Beijing, China; Department of Clinical Research and Data Management, Center of Respiratory Medicine, China-Japan Friendship Hospital, Beijing, China; Department of Pulmonary and Critical Care Medicine, Suzhou Municipal Hospital, The Affiliated Suzhou Hospital of Nanjing Medical University, Gusu School of Nanjing Medical University, Suzhou, China; Department of Pulmonary and Critical Care Medicine, China-Japan Friendship Hospital, Chinese Academy of Medical Sciences and Peking Union Medical College, Beijing, China; National Center for Respiratory Medicine; State Key Laboratory of Respiratory Health and Multimorbidity; National Clinical Research Center for Respiratory Diseases; Institute of Respiratory Medicine, Chinese Academy of Medical Sciences; Beijing Key Laboratory of Surveillance, Early Warning and Pathogen Research on Emerging Infectious Diseases; Laboratory of Clinical Microbiology and Infectious Diseases, Department of Pulmonary and Critical Care Medicine, Center of Respiratory Medicine, China-Japan Friendship Hospital, Beijing, China; Changping Laboratory, Beijing, China; National Center for Respiratory Medicine; State Key Laboratory of Respiratory Health and Multimorbidity; National Clinical Research Center for Respiratory Diseases; Institute of Respiratory Medicine, Chinese Academy of Medical Sciences; Beijing Key Laboratory of Surveillance, Early Warning and Pathogen Research on Emerging Infectious Diseases; Laboratory of Clinical Microbiology and Infectious Diseases, Department of Pulmonary and Critical Care Medicine, Center of Respiratory Medicine, China-Japan Friendship Hospital, Beijing, China; Beijing Taikang Yanyuan Rehabilitation Hospital, Beijing, China; Beijing Taikang Yanyuan Rehabilitation Hospital, Beijing, China; National Center for Respiratory Medicine; State Key Laboratory of Respiratory Health and Multimorbidity; National Clinical Research Center for Respiratory Diseases; Institute of Respiratory Medicine, Chinese Academy of Medical Sciences; Beijing Key Laboratory of Surveillance, Early Warning and Pathogen Research on Emerging Infectious Diseases; Laboratory of Clinical Microbiology and Infectious Diseases, Department of Pulmonary and Critical Care Medicine, Center of Respiratory Medicine, China-Japan Friendship Hospital, Beijing, China; Department of Pulmonary and Critical Care Medicine, China-Japan Friendship Hospital, Chinese Academy of Medical Sciences and Peking Union Medical College, Beijing, China; National Center for Respiratory Medicine; State Key Laboratory of Respiratory Health and Multimorbidity; National Clinical Research Center for Respiratory Diseases; Institute of Respiratory Medicine, Chinese Academy of Medical Sciences; Beijing Key Laboratory of Surveillance, Early Warning and Pathogen Research on Emerging Infectious Diseases; Laboratory of Clinical Microbiology and Infectious Diseases, Department of Pulmonary and Critical Care Medicine, Center of Respiratory Medicine, China-Japan Friendship Hospital, Beijing, China; New Cornerstone Science Laboratory, Beijing, China

**Keywords:** frailty, lower respiratory tract infection, older adults, risk factor

## Abstract

**Background:**

Lower respiratory tract infections (LRTIs) are a leading cause of morbidity in older adults, yet risk stratification based solely on chronological age remains insufficient. Frailty, reflecting cumulative declines in physiological reserve, may better capture susceptibility to respiratory infections. We aimed to investigate the association of frailty with the risk and frequency of LRTIs in older adults.

**Methods:**

We conducted a multicenter prospective cohort study among older adults in senior living communities in China, from November 2023 to September 2025. Baseline frailty was evaluated using the clinical frailty scale. Participants were monitored for LRTIs throughout the follow-up period. The association between frailty and LRTI risk was evaluated using cause-specific Cox proportional hazards models, with subgroup and sensitivity analyses performed to assess the consistency and robustness of the findings. For the LRTI frequency analysis, Poisson generalized estimating equation models were applied.

**Results:**

The cohort included 522 older adults with a median follow-up of 473.5 days. 104 LRTI episodes were documented, and 13.4% participants experienced at least one event. Frailty was a significant predictor of LRTI risk (adjusted HR, 1.29; 95% CI, 1.10–1.52), which remained robust across different LRTI types and sensitivity analyses. This association was particularly pronounced in participants with albumin levels < 45 g/L. Beyond initial infection risk, frailty was also associated with an increased frequency of LRTIs (adjusted incidence rate ratio, 1.30; 95% CI, 1.09–1.54).

**Conclusions:**

Frailty constitutes an independent and modifiable risk factor for LRTIs in older adults, emphasizing the importance of frailty management in LRTI preventive strategies.

Lower respiratory tract infection (LRTI) remains a leading cause of hospitalization and mortality among older adults [1]. According to the 1990–2021 burden of disease study in China, adults aged ≥ 70 years were most severely affected by non-COVID-19 LRTIs, with incidence and mortality rates of 16 231.1 and 138.9 per 100 000 population, respectively [2]. Currently, with the rapid aging of the population, the rising demand for LRTI-related hospitalization and critical care imposes a substantial burden on healthcare costs and resource utilization [3–5]. Although traditional risk factors for LRTIs, such as advanced age, heart failure, and chronic respiratory disease, are well established, their predictive value in older adults is limited [6]. These constraints arise largely from the high prevalence and considerable overlap of these factors within the geriatric population, which diminish their discriminative power for individual risk stratification. Therefore, identifying risk factors that better capture differences in susceptibility to LRTIs among older adults is important for improving risk assessment and guiding preventive interventions.

Frailty is an aging-related clinical syndrome characterized by declined physiological reserve and increased vulnerability to acute stressors [7, 8]. It is primarily manifested as extreme fatigue, slow gait, reduced physical activity, and unexplained weight loss [9]. In contrast to chronological age, frailty captures the heterogeneous and complex nature of biological aging and is strongly associated with adverse outcomes, including falls, disability, hospitalization, and death [10]. Interventions targeting this syndrome have also shown potential benefits in improving functional capacity and overall health status [11, 12]. Notably, frailty has been recognized as a major predictor of poor prognosis in pneumonia, such as ICU admission and 30-day mortality [13, 14]. However, evidence remains unclear on whether frailty itself contributes to the risk of developing LRTIs, highlighting the need for prospective research to clarify this relationship.

In this study, we aimed to assess the association between frailty and both the incidence risk (first episode) and frequency (recurrent episodes) of LRTIs in older adults, providing new insights to guide risk stratification and targeted prevention strategies for respiratory infection.

## METHODS

### Study Design and Participants

We conducted a prospective, observational, longitudinal cohort study across independent senior communities (Continuing Care Retirement Community, CCRC) in Beijing and Shanghai, China. CCRC is a comprehensive senior living community where each resident has complete medical documentation and benefits from ongoing health management provided by their general practitioners [15]. Older adults aged ≥ 60 years who consented to participate were recruited from November 1, 2023 to August 31, 2025. Exclusion criteria included: evidence of active infection at enrollment (infection-related symptoms or C-reactive protein ≥ 10 mg/L, or positive imaging findings) [16]; history of LRTIs within the past 3 months; history of solid organ or hematopoietic stem cell transplantation; HIV infection; terminal malignancy; life expectancy shorter than 6 months; or inability to complete questionnaires. The study followed the STROBE guidelines [17, 18] and was approved by the Clinical Research Ethics Committee of China-Japan Friendship Hospital (No. 2023-KY-066-2). All participants provided written informed consent at the time of admission.

### Data Collection and Frailty Assessment

Trained researchers specializing in geriatric health management collected the baseline data, including sociodemographic information (age, sex, body mass index [BMI], education level, marital status, and living arrangements), lifestyle factors (smoking, alcohol consumption, and secondhand smoke exposure), comorbidities, medication use, and vaccination history, specifically influenza vaccination within the past year and pneumococcal vaccination within 5 years. Additionally, complete blood count and serum albumin level from the first 24 hours postenrollment were also tested.

Frailty was assessed using the Clinical Frailty Scale (CFS), a validated tool originally developed in a large cohort of Canadian older adults, which numerically ranks frailty severity from 1 (very fit) to 9 (terminally ill) [19]. It can be easily implemented in clinical settings, demonstrates good inter-rater reliability, and correlates strongly with the 70-item Frailty Index [20]. In this study, the frailty level for each participant was evaluated according to their basic activities of daily living, instrumental activities of daily living, number of chronic conditions, physical strength, and self-rated health. The CFS score was primarily treated as a continuous variable, with secondary analyses using categorical cutoffs established in prior research [21, 22].

### Follow-up and Outcomes

Participants were prospectively followed until 30 September 2025. Daily monitoring was performed using hospital visit records and pharmacy prescription data, supplemented by structured in-person follow-up visits every 2 weeks to identify any new respiratory symptoms or signs suggestive of LRTIs. All reported clinical events were verified against medical records and confirmed by respiratory specialists. Based on the 2011 ERS/ESCMID Guidelines for the Management of Adult Lower Respiratory Tract Infections [23], participants were diagnosed with LRTIs if they met the following criteria: (1) acute cough lasting ≤ 21 days, with at least one lower respiratory symptom, such as sputum production, dyspnea, or chest discomfort/pain; (2) abnormal white blood cell count or differential [24]; (3) new pulmonary infiltrates on chest imaging, or significant radiographic progression of pre-existing pulmonary lesions [25]; (4) exclusion of other causes such as sinusitis or asthma attack. Available microbiological findings and clinical outcomes for LRTIs were also collected from the medical records. After incident LRTIs were identified, each episode was further classified into different clinical types using published criteria, including acute bronchitis (AB), community-acquired pneumonia (CAP), acute exacerbation of chronic obstructive pulmonary disease (AECOPD), and acute exacerbation of bronchiectasis (AEBX). Detailed definitions are provided in the Supplementary Methods. The primary outcome of this study was the risk of first LRTI occurrence, and the secondary outcome was the frequency of LRTI episodes throughout the follow-up period.

### Statistical Analysis

Continuous variables were summarized as mean (standard deviation) for normally distributed data and as median (interquartile range [IQR]) for skewed data, while categorical variables were presented as counts and percentages. Baseline characteristics were compared using Student's *t*-test for normally distributed variables, and the Mann–Whitney *U* test for non-normal variables. Differences in categorical variables and LRTI frequency were analyzed with the χ^2^ or Fisher exact test, as appropriate. Median imputation was performed for 2 participants with missing laboratory test results before analysis.

For the primary analysis, the cumulative incidence of LRTIs across frailty subgroups was first estimated using the cumulative incidence function (CIF) to account for the competing risk of death, and compared using Gray's test. A multivariable cause-specific Cox proportional hazards model was employed to quantify the association between frailty and LRTI risk, adjusting for potential confounding factors: demographics (age, sex, and BMI), lifestyles (smoking, drinking, and secondhand smoke exposure), comorbidities (heart failure, diabetes, COPD, bronchiectasis, stroke, solid tumor), immunosuppressives, vaccination history, and albumin. Results were reported as adjusted hazard ratios (aHRs) and 95% confidence intervals (CIs). Model assumptions were verified by examining Schoenfeld residuals for proportional hazards and variance inflation factors (VIFs) for multicollinearity. We also applied the restricted cubic spline (RCS) function with 4 knots to explore any potential nonlinear relationship between continuous CFS score and LRTI risk [26]. Sensitivity analyses were performed to assess the robustness of the primary results (see the Supplementary Methods).

Subgroup analyses were conducted using the multivariable cause-specific Cox proportional hazards model to evaluate the consistency of the frailty-LRTI association across different populations. Participants were stratified by age (<80 vs ≥ 80 years), sex (female vs male), BMI (<24 vs ≥ 24 kg/m^2^), smoking status (never vs former/current), drinking status (never vs former/current), chronic respiratory disease (yes vs no), and albumin levels (< 45 vs ≥ 45 g/L). Risk estimates for each subgroup were adjusted for all covariates except the stratifying variable and reported as aHRs with 95% CIs. Interactions between frailty and these stratification variables were also examined.

For the secondary outcome regarding LRTI frequency, the cumulative burden of recurrent events was characterized using the mean cumulative function (MCF). Subsequently, we applied a multivariable Poisson regression model with generalized estimating equations (GEE) to evaluate the independent impact of frailty on LRTI frequency. The model was adjusted for the covariates described above, with results presented as incidence rate ratios (IRRs) and 95% CIs.

Additionally, we extended our analysis to explore the associations of frailty with different LRTI types, as well as subsequent clinical outcomes. Detailed methodologies for these supplementary analyses are described in the Supplementary Methods. All statistical analyses were performed using IBM SPSS version 27.0 and R version 4.4.1. Two-sided *P* values < .05 were considered statistically significant.

## RESULTS

### Baseline Demographic and Clinical Characteristics

**Figure 1.**
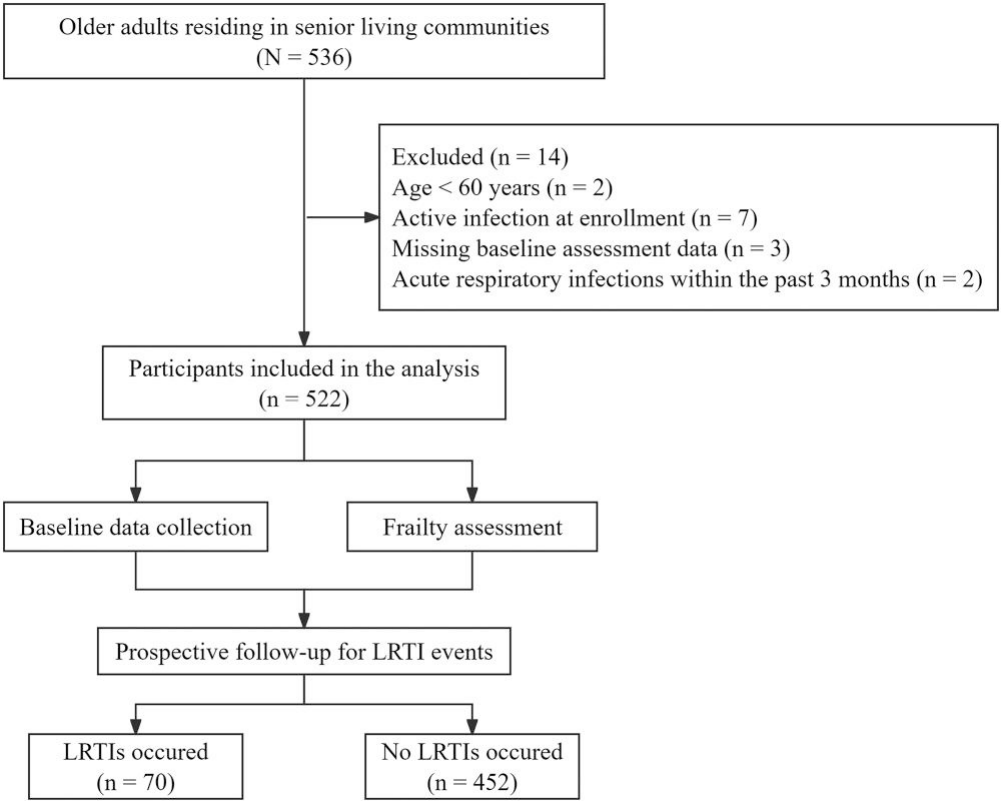
Flowchart of the participant selection process. LRTIs, lower respiratory tract infections.

A total of 536 older adults were enrolled, of whom 14 were excluded for not fulfilling the inclusion/exclusion criteria (Figure 1). The final analysis included 522 participants, with a median follow-up of 473.5 days (IQR, 330.5–589.0), and 7 individuals died from causes other than respiratory infections. Baseline clinical characteristics of participants are presented in Table 1. The cohort was predominantly female (60.7%) with a median age of 82 years (IQR, 76–87) and a median CFS score of 3 (IQR, 2–5). Based on previous established definitions [27], 65.1% of participants (*n* = 340) with CFS score 1–4 were classified as nonfrail, and 182 (34.9%) with CFS score 5–9 as frail. Compared with the nonfrail group, participants with frailty were older (83 vs 80 years, *P* < .01) and had a greater burden of comorbidities, including cardiovascular disease, COPD, renal disease, Alzheimer's disease, Parkinson's disease, and stroke. They also had lower lymphocyte (1.70 vs 1.84 × 10^9^/L, *P* < .01), red blood cell (4.20 vs 4.48 × 10^12^/L, *P* < .001), hemoglobin (132 vs 140 g/L, *P* < .001), and albumin levels (41.78 vs 43.99 g/L, *P* < .001).

**Table 1.** Baseline Clinical Characteristics of Participants by Frailty Status

Variables	Total (*N* = 522)	CFS 1–4 (*N* = 340)	CFS 5–9 (*N* = 182)	*P* Value
Age, years	82.00 [76.00, 87.00]	80.00 [76.00, 86.00]	83.00 [77.00, 88.25]	<.01
Sex, *n* (%)				.45
Male	205 (39.27)	138 (40.59)	67 (36.81)	
Female	317 (60.73)	202 (59.41)	115 (63.19)	
BMI, kg/m^2^	23.60 [21.68, 25.80]	23.56 [21.70, 25.59]	23.90 [21.50, 26.23]	.44
Education level, *n* (%)				.95
College education or above	398 (76.25)	260 (76.47)	138 (75.82)	
High school education or lower	124 (23.75)	80 (23.53)	44 (24.18)	
Marital status, *n* (%)				.04
Married	349 (66.86)	238 (70.00)	111 (60.99)	
Widowed/divorced/single	173 (33.14)	102 (30.00)	71 (39.01)	
Living arrangements, *n* (%)				.81
Living alone	180 (34.48)	119 (35.00)	61 (33.52)	
Living with others	342 (65.52)	221 (65.00)	121 (66.48)	
Smoking status, *n* (%)	71 (13.60)	47 (13.82)	24 (13.19)	.95
Secondhand smoke, *n* (%)	286 (54.79)	177 (52.06)	109 (59.89)	.11
Drinking status, *n* (%)	118 (22.61)	82 (24.12)	36 (19.78)	.31
Comorbidities, *n* (%)				
Heart failure	38 (7.28)	12 (3.53)	26 (14.29)	<.01
Hypertension	349 (66.86)	214 (62.94)	135 (74.18)	.01
Diabetes	143 (27.39)	88 (25.88)	55 (30.22)	.34
Bronchiectasis	26 (4.98)	18 (5.29)	8 (4.40)	.81
Asthma	29 (5.56)	20 (5.88)	9 (4.95)	.81
COPD	37 (7.09)	18 (5.29)	19 (10.44)	.04
ILD	32 (6.13)	16 (4.71)	16 (8.79)	.10
OSAS	19 (3.64)	11 (3.24)	8 (4.40)	.67
GERD	170 (32.57)	108 (31.76)	62 (34.07)	.66
Chronic liver disease	17 (3.26)	11 (3.24)	6 (3.30)	>.90
Renal disease	46 (8.81)	19 (5.59)	27 (14.84)	<.01
Alzheimer's disease	30 (5.75)	1 (0.29)	26 (14.29)	<.01
Parkinson's disease	8 (1.53)	2 (0.59)	6 (3.30)	.02
Stroke	88 (16.86)	42 (12.35)	46 (25.27)	<.01
Solid tumor	89 (17.05)	60 (17.65)	29 (15.93)	.71
Immunosuppressives, *n* (%)	10 (1.92)	6 (1.76)	4 (2.20)	.75
Influenza vaccination	251 (48.08)	156 (45.88)	95 (52.20)	.20
Pneumococcal vaccination	222 (42.53)	140 (41.18)	82 (45.05)	.40
Laboratory test				
WBC, × 10^9^/L	5.60 [4.72, 6.60]	5.60 [4.78, 6.60]	5.62 [4.62, 6.73]	.74
Neutrophil count, × 10^9^/L	3.14 [2.55, 3.99]	3.12 [2.55, 3.94]	3.21 [2.52, 4.10]	.48
Lymphocyte count, × 10^9^/L	1.78 [1.42, 2.24]	1.84 [1.45, 2.32]	1.70 [1.34, 2.12]	<.01
RBC, × 10^12^/L	4.41 [4.08, 4.72]	4.48 [4.21, 4.83]	4.20 [3.94, 4.53]	<.01
Hemoglobin, g/L	136.00 [128.00, 145.00]	140.00 [131.00, 148.00]	132.00 [122.00, 140.00]	<.01
PLT, × 10^9^/L	189.00 [161.00, 225.00]	192.00 [167.00, 227.75]	181.50 [148.00, 216.00]	<.01
ALB, g/L	43.25 [41.27, 45.15]	43.99 [42.03, 45.79]	41.78 [39.80, 44.22]	<.01
LRTI, *n* (%)	70.00 (13.41)	32.00 (9.41)	38.00 (20.88)	<.01
AB	1.00 (0.19)	1.00 (0.29)	0.00 (0.00)	>.90
CAP	58.00 (11.11)	27.00 (7.94)	31.00 (17.03)	<.01
AEBX	3.00 (0.57)	2.00 (0.59)	1.00 (0.55)	>.90
AECOPD	8.00 (1.53)	2.00 (0.59)	6.00 (3.30)	.02
Number of LRTI episodes, *n* (%)				<.01
0	452 (86.59)	308 (90.59)	144 (79.12)	
1	50 (9.58)	24 (7.06)	26 (14.29)	
2	12 (2.30)	5 (1.47)	7 (3.85)	
3	3 (0.57)	1 (0.29)	2 (1.10)	
4	4 (0.77)	2 (0.59)	2 (1.10)	
5	1 (0.19)	0 (0.00)	1 (0.54)	
Follow-up time, days	473.50 [330.50, 589.00]	463.00 [322.25, 603.00]	511.00 [395.00, 583.50]	.21

Values are represented in mean (standard deviation), median [Q1, Q3], or *n* (%). Chronic liver disease includes viral hepatitis and liver cirrhosis. Renal disease includes chronic glomerulonephritis, nephrotic syndrome, and chronic renal failure.

AB, acute bronchiectasis; AEBX, acute exacerbation of bronchiectasis; AECOPD, acute exacerbation of chronic obstructive pulmonary disease; ALB, albumin; BMI, body mass index; CAP, community-acquired pneumonia; CFS, clinical frailty scale; COPD, chronic obstructive pulmonary disease; GERD, gastroesophageal reflux disease; ILD, interstitial lung disease; LRTI, lower respiratory tract infection; OSAS, obstructive sleep apnea syndrome; PLT, platelet; RBC, red blood cell; WBC, white blood cell.

In the entire cohort, 70 participants (13.4%) had at least one LRTI episode during follow-up. Participants in the frail group exhibited both a greater proportion (20.9% vs 9.4%, *P* < .001) and frequency (*P* < .01) of LRTIs than the nonfrail group, which provided preliminary evidence that LRTIs were more common among frail individuals (Table 1). 104 LRTI episodes were documented during the follow-up, of which 68.3% underwent at least one form of microbiologic testing, including rapid viral antigen tests, nucleic acid detection, and conventional sputum cultures. Pathogens were identified in 62.0% of those tested, and bacteria were the most frequently detected (29.6%), with *Streptococcus pneumoniae* and *Klebsiella pneumoniae* being the predominant species. Notably, no significant differences were observed in any specific pathogen category between the frail and nonfrail groups (all *P* values > .05) (Supplementary Table 1).

### Association Between Frailty and LRTI Risk

**Figure 2.**
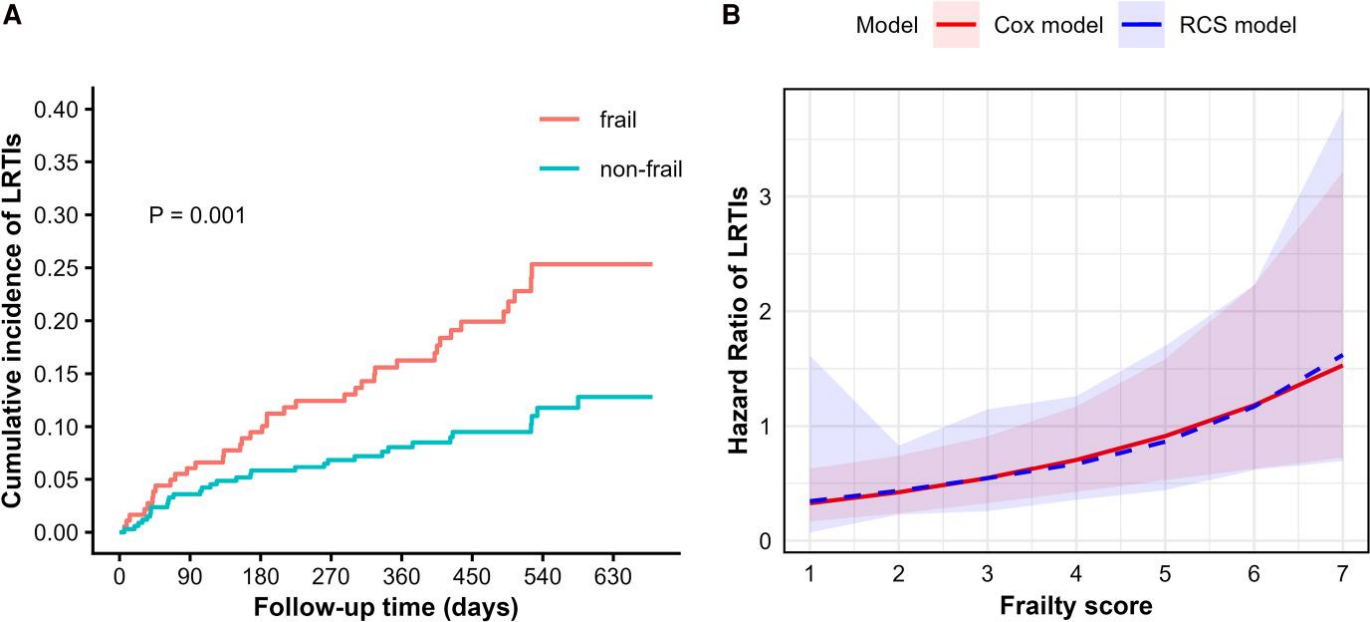
Association between frailty and the risk of LRTIs. *A*, Cumulative incidence of LRTIs in frail and nonfrail groups. *P* value was calculated using Gray's test. *B*, Association between frailty and LRTI risk based on cause-specific Cox regression models, with and without RCS function. Models were adjusted for age, sex, body mass index, smoking status, secondhand smoke exposure, drinking status, heart failure, diabetes, bronchiectasis, chronic obstructive pulmonary disease, stroke, solid tumor, immunosuppressives, vaccination history, and serum albumin levels. CFS, clinical frailty scale; LRTIs, lower respiratory tract infections; RCS, restricted cubic spline.

Regarding the primary outcome, CIF analysis indicated that frail participants had a significantly higher cumulative incidence of LRTIs relative to their nonfrail counterparts (Gray's test, *P* = .001, Figure 2*A*). Considering death as a competing event for LRTI occurrence, we performed a multivariable cause-specific Cox regression model. The proportional hazards assumption was satisfied (Global *P* = .71; Supplementary Figure 1), and no substantial multicollinearity was observed among the covariates (all VIFs < 2; Supplementary Table 2). Notably, frailty was independently associated with an increased risk of LRTIs (aHR, 1.29; 95% CI, 1.10–1.52). This association remained robust across all sensitivity analyses, including the use of the Fine-Gray competing risk model and exclusions of specific populations (Supplementary Table 3). In the RCS analysis with 4 knots, we further investigated the dose-response relationship between the continuous CFS score and LRTI risk (Figure 2*B*). The model revealed an overall significant positive association (Wald test, *P* = .02), while the test for nonlinearity was not significant (ANOVA, *P* = .94). These results indicated that the risk of LRTIs increased in a consistent, linear fashion with worsening frailty, rather than following a more complex curve or having the threshold effect.

### Subgroup and Interaction Analyses

**Figure 3.**
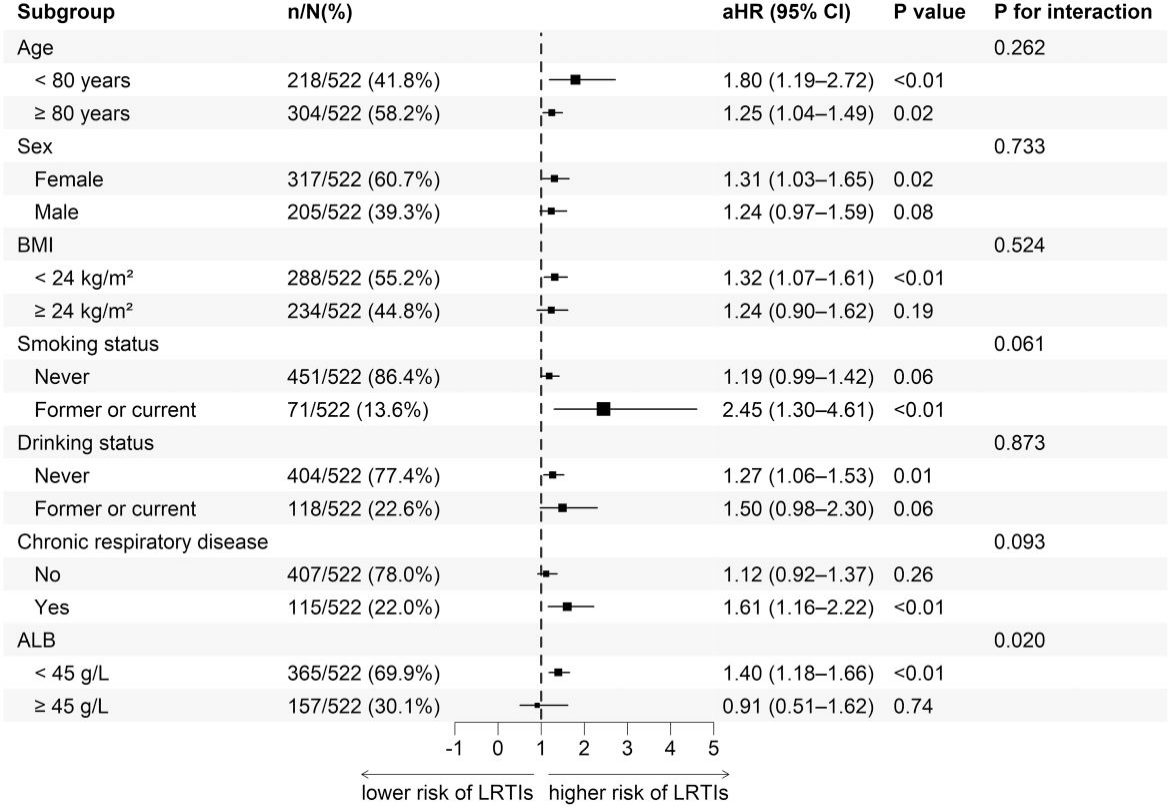
Subgroup analyses and interaction tests for the association of frailty with LRTI risk. The position of each square represents the point estimate of the adjusted HR for frailty, and the horizontal lines represent the 95% confidence intervals. The vertical dashed line at 1 indicates the reference value of no effect. All analyses were performed using multivariable cause-specific Cox regression models, adjusted for all confounding factors except the variable used for stratification. aHR, adjusted hazard ratio; CI, confidence interval; ALB, albumin; BMI, body mass index; LRTIs, lower respiratory tract infections.

We conducted subgroup analyses stratified by age, sex, BMI, smoking status, drinking status, chronic respiratory disease, and albumin levels. The relationship between frailty and LRTI risk was consistent across most strata (all *P* for interaction > .05), with the notable exception of serum albumin (Figure 3). Specifically, frailty was significantly associated with LRTI risk in participants with low-albumin levels (<45 g/L) (*P* < .01), whereas this association failed to reach statistical significance in the high-albumin subgroup (≥45 g/L) (*P* = .74). Interaction analysis further confirmed that albumin significantly modified the frailty-LRTI association when treated as a continuous variable (*P* for interaction = .02).

### Association Between Frailty and LRTI Frequency

**Figure 4.**
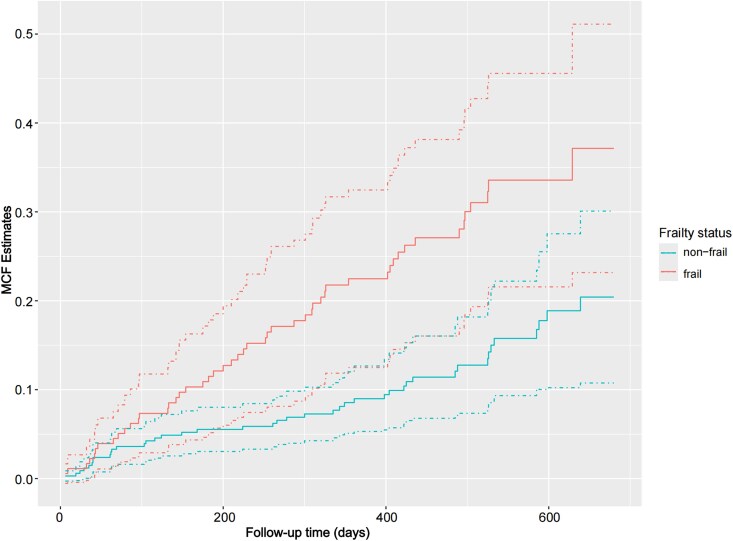
MCF of recurrent LRTI episodes in frail and nonfrail groups. The curves represent the estimated mean cumulative number of lower respiratory tract infections. The solid lines indicate the point estimates, and the dashed lines represent the 95% confidence intervals. CFS, clinical frailty scale; MCF, mean cumulative function.

Approximately 28.6% of participants who developed LRTIs in this cohort experienced 2 or more episodes during the observation period. The overall incidence rate of LRTIs was 15.94 per 100 person-years, substantially exceeding the national epidemiological data reported in China between 1990 and 2021 [28]. As illustrated by the MCF curves (Figure 4), the frail group exhibited a consistently steeper trajectory compared with the nonfrail group, indicating a more rapid accumulation of LRTI events over time. Consistent with the MCF analysis, multivariable Poisson-GEE models also confirmed that frailty was independently associated with a higher frequency of developing LRTIs (adjusted IRR, 1.30; 95% CI, 1.09–1.54).

### Association of Frailty With LRTI Types and Prognosis

In additional exploratory analyses, frailty was associated with an increased risk of CAP (aHR, 1.34; 95% CI, 1.12–1.60). Among participants who developed LRTIs, frail individuals were more frequently hospitalized than nonfrail individuals (52.5% vs 26.7%, *P* = .01), more often required high-flow nasal cannula therapy (30.5% vs 2.2%, *P* < .001), and had a longer clinical course (14 vs 11 days, *P* < .01). Detailed results are provided in the Supplementary Results.

## DISCUSSION

In this prospective cohort study, we demonstrated that frailty was an independent risk factor for the development of LRTIs in older adults. Frailty was associated not only with the incidence of initial LRTIs but also the frequency of recurrent infections. Notably, the frailty-LRTI risk association appeared to be modified by nutritional status, which was particularly pronounced in individuals with low-albumin levels. Moreover, beyond the heightened susceptibility to respiratory infections, frail individuals tended to experience more adverse outcomes following LRTI onset. To our knowledge, this is the first multicenter prospective study to evaluate the contribution of frailty to the overall burden of LRTIs, providing new perspectives for identifying and managing older adults at higher risk of infection.

Consistent with previous studies, our findings support a strong association between frailty and increased susceptibility to LRTIs in older adults [29, 30]. However, most existing evidence is derived from cross-sectional or retrospective studies in which frailty was assessed during active infection or hospitalization, making it challenging to distinguish pre-existing vulnerability from infection-induced physiological stress responses [31, 32]. By contrast, frailty in our study was evaluated at baseline before the onset of any acute illness, thereby minimizing reverse causation and enhancing the interpretability of its association with LRTIs. The underlying pathophysiological mechanisms linking frailty to LRTIs are multifactorial, including immunosenescence, chronic low-grade inflammation, reduced airway clearance, swallowing dysfunction, and metabolic alterations [7, 31]. These combined physiological impairments substantially contribute to the onset and recurrence of LRTIs. Taken together, frailty represents an important host factor that confers persistent vulnerability to respiratory insults in the aging population.

The association between frailty and LRTI risk was generally consistent across most subgroups, which reinforced the robustness and reliability of our main results. However, statistical significance was not observed in certain groups, including males, never-smokers, individuals without chronic respiratory disease, and those with BMI ≥ 24 kg/m^2^. This lack of significance may reflect limited sample size reducing statistical power, and it could also suggest that frailty exerts its greatest influence at the extremes of physiological vulnerability. An important discovery from our analysis was the significant effect modification by nutritional status, where the frailty-LRTI association was more pronounced among individuals with albumin < 45 g/L. Although malnutrition is traditionally defined as < 35 g/L in clinical practice [33], our data indicate that even albumin levels within the lower physiological range may amplify infection risk in frail older adults. This likely represents a “second hit” phenomenon where modest albumin reductions further compromise pulmonary defenses in frail individuals already affected by immunosenescence and chronic inflammation [34, 35]. Therefore, integrating nutritional assessment and support is crucial for managing infection risk in this vulnerable population.

Our research corroborates and extends the understanding that frailty, as the comprehensive measure of biological aging, is a more critical determinant of LRTI risk in older adults than chronological age alone. In univariate Cox models, frailty demonstrated superior predictive power with a HR of 1.38 compared with 1.06 for age, and this association remained robust across all age subgroups. Importantly, unlike chronological age, frailty is a modifiable condition that can be improved or even reversed through effective strategies such as physical exercise, cognitive training, and polypharmacy management [36–38]. Collectively, our results highlight the importance of considering frailty not merely as a biomarker of overall health decline, but as a potential target for early risk stratification and preventive interventions, which could help alleviate the disproportionate burden of LRTIs in elderly populations.

A major strength of this study lies in its multicenter, prospective cohort design, which enabled us to capture temporal changes and establish a clear causal link between pre-existing frailty and subsequent LRTIs across different populations. The use of the standardized CFS at baseline enhanced comparability across participants from different study sites, while the comprehensive collection of demographic, clinical, functional, and laboratory data facilitated adjustment for a wide range of potential confounders. Moreover, the application of multiple complementary statistical models strengthened the robustness of findings and minimized the likelihood of bias.

Several limitations should be considered. First, participants were exclusively recruited from CCRCs, a relatively selected population with more congregate living environments and closer health monitoring than community-dwelling older adults. Although the stable environment with continuous care facilitated more complete follow-up, these differences in population and residential features may influence LRTI risk and patterns of pathogen exposure, thereby limiting the generalizability of our findings to older adults living in more heterogeneous settings. Second, frailty was assessed only at baseline, which may not fully capture its dynamic nature during follow-up. This could have introduced exposure misclassification and attenuated the observed association toward the null, potentially underestimating the true impact of frailty on LRTI development. Third, LRTIs were primarily confirmed based on clinical and radiologic criteria, and microbiologic information was available for only 68.3% of infection episodes, which limited the completeness of etiologic assessment. Finally, the modest sample size may have reduced statistical power in subgroup and prognostic analyses, increasing the risk of imprecise estimates and false-negative findings. Future larger-scale studies with repeated frailty assessments are warranted to confirm these results and explore potential heterogeneity across diverse populations.

## CONCLUSION

Frailty serves as an important predictor of both the risk and frequency of LRTIs in older adults. Early identification and targeted interventions for frailty may help mitigate the burden of respiratory infections. Our longitudinal study bridges the gap between observational research and practical clinical application, providing new insights into LRTI risk factors and highlighting frailty as a treatable trait in this vulnerable population. Further research should focus on elucidating the lung-specific effects of frailty and its underlying biological mechanisms, thereby facilitating the development of innovative treatments to protect older people from respiratory disease.

## Supplementary Material

ofag223_Supplementary_Data

## Supplementary Data

Supplementary materials are available at *Open Forum Infectious Diseases* online. Consisting of data provided by the authors to benefit the reader, the posted materials are not copyedited and are the sole responsibility of the authors, so questions or comments should be addressed to the corresponding author.
