# Supplementary material for: Frailty Increases the Risk and Frequency of Lower Respiratory Tract Infections in Older Adults: A Multicenter Prospective Cohort Study
Source: Open Forum Infect Dis. 2026 Apr 24;13(5):ofag223. doi: 10.1093/ofid/ofag223 (PMC13123861; doi:10.1093/ofid/ofag223)
Supplement: ofag223_Supplementary_Data [file ofag223_supplementary_data.doc]

**Frailty Increases the Risk and Frequency of Lower Respiratory Tract Infections in Older Adults: A Multicenter Prospective Cohort Study**

Supplementary document

**Supplementary Methods**

**Diagnostic Definitions of LRTI Types**

All outcome events were first identified as incident lower respiratory tract infections (LRTIs). Each episode was then classified into specific types according to the clinical manifestations and published guidelines as follows.

(1) acute bronchitis (AB): acute cough lasting < 3 weeks, without chronic lung disease or new infiltrates on chest radiography [1].

(2) community-acquired pneumonia (CAP): newly recognized pulmonary infiltrate on chest imaging, together with at least one respiratory symptom (e.g., cough, sputum production, chest pain, dyspnea, or hemoptysis) and at least one additional sign or finding, such as fever, abnormal lung sounds, hypoxemia, or an abnormal peripheral white blood cell count (> 10 × 10^9^/L or < 4 × 10^9^/L) [2,3].

(3) acute exacerbation of chronic obstructive pulmonary disease (AECOPD): acute worsening of respiratory symptoms (cough, dyspnea, and/or sputum production) in a patient with a previous diagnosis of COPD, warranting a change in management [4].

(4) acute exacerbation of bronchiectasis (AEBX): deterioration in three or more key symptoms for at least 48 hours in a patient with a previous diagnosis of bronchiectasis, including cough, sputum volume and/or consistency, sputum purulence, dyspnea, hemoptysis, and fatigue or malaise, requiring a change in treatment [5,6].

**Statistical Analysis**

Sensitivity analyses were performed to assess the robustness of the association between frailty and LRTI risk. First, we conducted a Fine-Gray sub-distribution hazard model to account for the competing risk of death. Second, we repeated the main cause-specific Cox regression models after removing individuals with incomplete baseline data. Furthermore, we tested potential reverse causality by excluding participants who developed LRTIs within the first 90 days of follow-up. Finally, we also right-censored the follow-up at 540 days to mitigate the potential impact of the time-varying nature of frailty while ensuring adequate statistical power.

Considering the heterogeneity of LRTIs observed in our cohort, cumulative incidence function (CIF) curves were constructed for different types of LRTIs to visualize the type-specific incidence across frailty subgroups over time. We also performed multivariable cause-specific Cox proportional hazards analyses to estimate the association between frailty and the risk of LRTI types. In these analyses, death and LRTI events other than the type of interest were treated as competing events. Covariate adjustment remained consistent with the primary Cox analysis, and results were reported as adjusted hazard ratios (aHRs) with corresponding 95% confidence intervals (CIs).

We employed generalized estimating equation (GEE) models to investigate the association between frailty and the clinical prognosis of LRTIs, accounting for the correlation of repeated events within individuals. Specific endpoints included hospitalization, high-flow nasal cannula (HFNC) therapy, mechanical ventilation, intensive care unit (ICU) admission, clinical course, and death. A gamma distribution with a log link was applied for the duration of illness, with results reported as mean ratios and 95% CIs, whereas a binomial distribution with a logit link was used for binary outcomes, with results reported as odds ratios (ORs) and 95% CIs. Due to the limited number of clinical outcomes, unadjusted models were used to avoid overfitting.

**Supplementary Results**

**Sensitivity Analyses**

In the sensitivity analyses, the association between frailty and LRTI risk remained significant when analyzed using the Fine-Gray competing risk model. Results were consistent with the primary analysis after removing individuals with incomplete baseline data. Additionally, findings remained robust in analyses excluding participants who developed LRTIs within 90 days or censoring follow-up at 540 days (**Supplementary Table S3**).

**Association Between Frailty and LRTI Types**

Among the 70 first episodes of incident LRTIs, 82.9% were CAP (**Table 1**). Given the limited number of non-pneumonia events (n = 12), LRTI episodes were classified into two broader categories: pneumonia (all cases were CAP in this cohort) and non-pneumonia LRTIs (AB, AEBX, and AECOPD). Frail participants exhibited a higher cumulative incidence of pneumonia than non-frail participants (Gray’s test, *P* = 0.006, **Supplementary Figure S2A**). This association remained significant in the Cox analysis, with frailty associated with an increased risk of pneumonia (aHR, 1.34; 95% CI, 1.12-1.60). In contrast, no significant relationship was observed between frailty and the risk of non-pneumonia LRTIs in either Gray’s test (*P* = 0.10, **Supplementary Figure S2B**) or Cox models (aHR, 1.07; 95% CI, 0.69-1.65), possibly attributable to insufficient statistical power resulting from the small number of events.

**Association Between Frailty and LRTI Prognosis**

Multiple clinical outcomes of LRTIs were compared between the frail and non-frail groups (**Supplementary Table S1**). Frail participants had higher rates of hospitalization (52.5% vs 26.7%, *P* = 0.01) and HFNC therapy (30.5% vs 2.2%, *P* = 0.04), and experienced a longer clinical course than non-frail individuals (14 vs 11 days, *P* < 0.01). In contrast, no significant differences were observed in ICU admission, mechanical ventilation, or death.

In the GEE analyses treating frailty as a continuous variable, the Gamma regression model showed that frailty was significantly associated with a longer clinical course of LRTIs. Specifically, each one-point increase in Clinical Frailty Scale (CFS) score was associated with a 19% increase in the mean duration of illness (Exp (beta) = 1.19; 95% CI, 1.06-1.33). In addition, GEE logistic models also revealed that greater frailty severity was independently associated with increased odds of hospitalization (OR, 1.49; 95% CI, 1.13-1.97) and HFNC therapy requirement (OR, 2.66; 95% CI, 1.27-5.57).


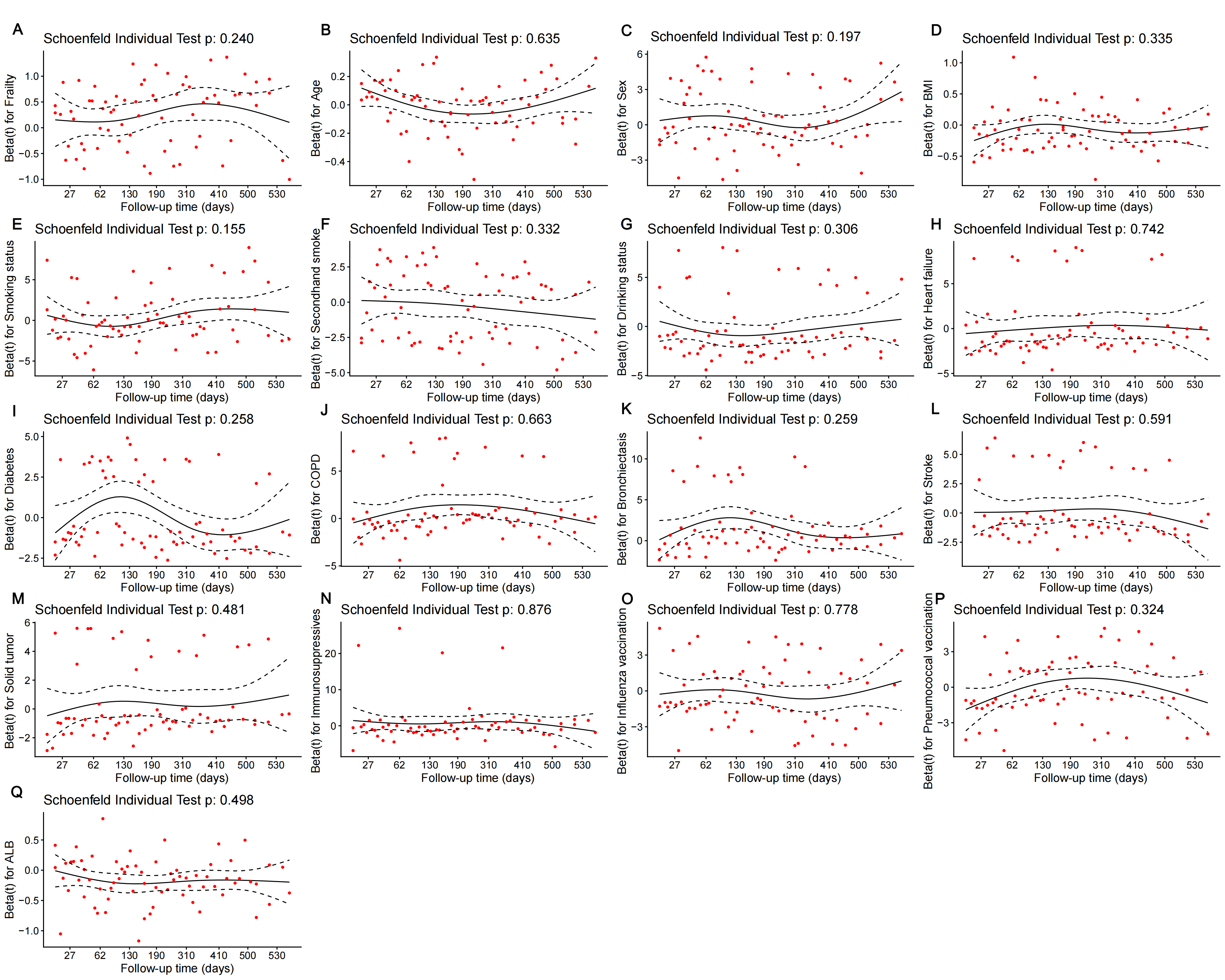


**Figure S1**. Assessment of the proportional hazards assumption in the main multivariable cause-specific Cox model. Scaled Schoenfeld residuals plotted against transformed time for each covariate included in the main multivariable cause-specific Cox model. The solid line represents the smoothing spline fit, and the dashed lines represent the ±2 standard error bands. A horizontal trend line indicates that the proportional hazards assumption is satisfied. Non-significant P-values (all P > 0.05) for individual covariates and the global test indicate no violation of the proportional hazards assumption. Panels (A) to (Q) correspond to: (A) Frailty, (B) Age, (C) Sex, (D) BMI, (E) Smoking status, (F) Secondhand smoke, (G) Drinking status, (H) Heart failure, (I) Diabetes, (J) COPD, (K) Bronchiectasis, (L) Stroke, (M) Solid tumor, (N) Immunosuppressives, (O) Influenza vaccination, (P) Pneumococcal vaccination, (Q) ALB. ALB, albumin; COPD, chronic obstructive pulmonary disease.


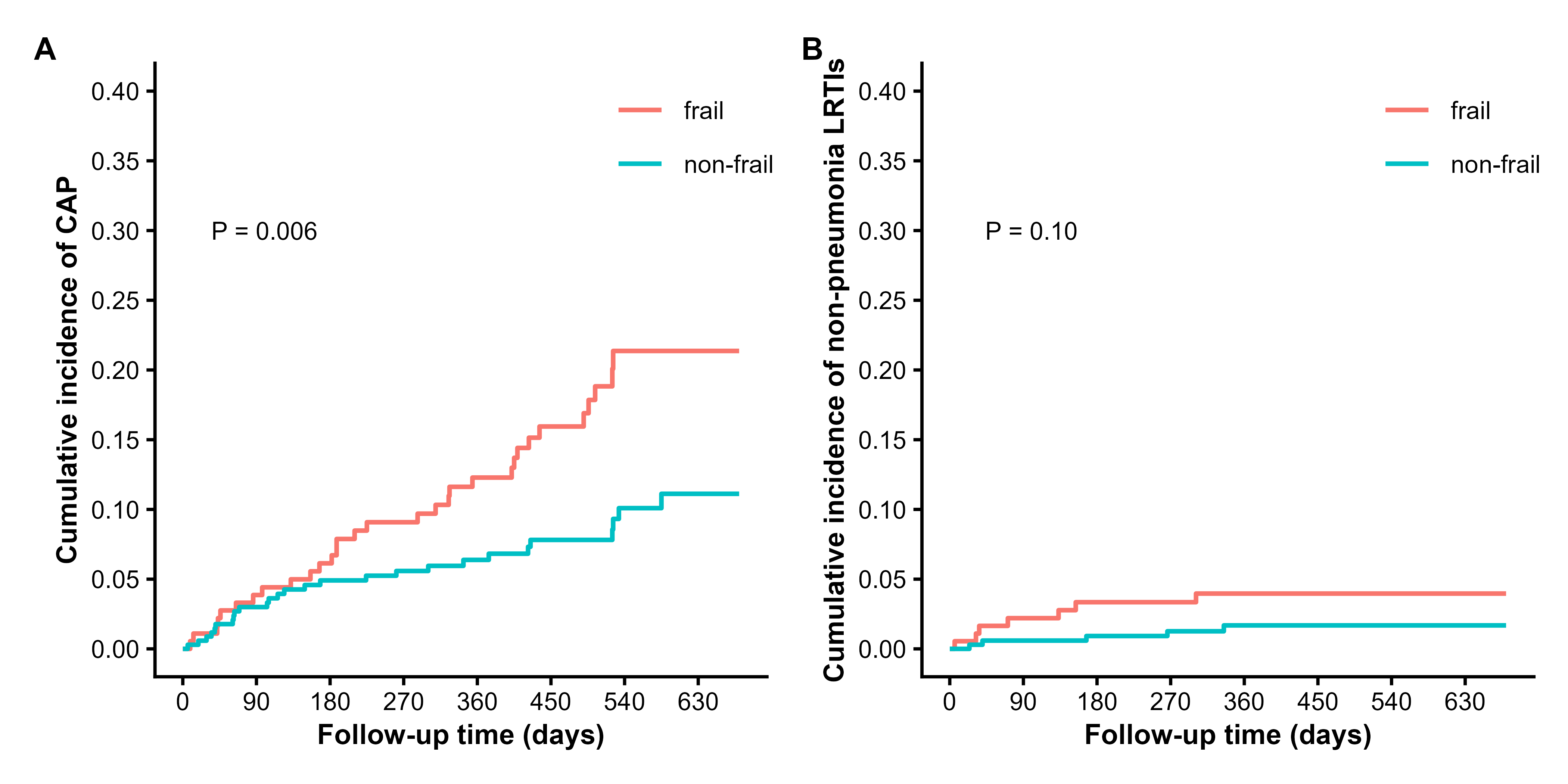


**Figure S2**. Association between frailty and the risk of LRTI types. (A) Cumulative incidence of pneumonia in frail and non-frail groups. (B) Cumulative incidence of non-pneumonia LRTIs in frail and non-frail groups. P value was calculated using Gray’s test. LRTIs, lower respiratory tract infections.

**Table S1.** Microbiological findings and clinical outcomes of LRTIs by frailty status

| Variables | Total (N = 104) | CFS 1–4 (N = 45) | CFS 5–9 (N = 59) | *P* value |
| --- | --- | --- | --- | --- |
| Microbiological testing, n (%) | 71 (68.27) | 28 (62.22) | 43 (72.88) | 0.45 |
| Bacteria | 21 (29.58) | 9 (32.14) | 12 (27.91) | 0.76 |
| Viruses | 11 (15.49) | 7 (25.00) | 4 (9.30) | 0.09 |
| Atypical pathogens | 6 (8.45) | 2 (7.14) | 4 (9.30) | 0.71 |
| Mixed infections | 6 (8.45) | 2 (7.14) | 4 (9.30) | 0.67 |
| No pathogen detected | 27 (38.03) | 8 (28.57) | 19 (44.19) | 0.20 |
| Clinical outcomes | | | | |
| Hospitalization, n (%) | 43 (41.35) | 12 (26.67) | 31 (52.54) | 0.01 |
| HFNC therapy, n (%) | 19 (18.27) | 1 (2.22) | 18 (30.51) | 0.04 |
| Mechanical ventilation, n (%) | 5 (4.81) | 1 (2.22) | 4 (6.78) | 0.45 |
| ICU admission, n (%) | 3 (2.88) | 1 (2.22) | 2 (3.39) | 0.67 |
| Duration of illness, days | 12.00 [9.00, 18.00] | 11.00 [8.00, 17.00] | 14.00 [9.00, 23.00] | <0.01 |
| Death, n (%) | 6 (5.77) | 1 (2.22) | 5 (8.47) | 0.19 |
| Note: Data are presented as n (%) or median [Q1, Q3]. Mixed infections were defined as the simultaneous detection of two or more pathogens in a single LRTI episode. Percentages for different pathogens were calculated using the incident LRTI episodes with microbiologic testing as the denominator.  Abbreviations: CAP, community-acquired pneumonia; AB, acute bronchitis; AEBX, acute exacerbation of bronchiectasis; AECOPD, acute exacerbation of chronic obstructive pulmonary disease; CFS, Clinical Frailty Scale; HFNC, high-flow nasal cannula; ICU, intensive care unit; LRTIs, lower respiratory tract infections. | | | | |

**Table S2.** Variance inflation factors for covariates in the multivariable cause-specific Cox regression model

| Covariates | VIF |  |
| --- | --- | --- |
| Frailty | 1.41 |  |
| Age | 1.42 |  |
| Sex | 1.51 |  |
| BMI | 1.21 |  |
| Smoking status | 1.53 |  |
| Secondhand smoke | 1.20 |  |
| Drinking status | 1.23 |  |
| Heart failure | 1.17 |  |
| Diabetes | 1.08 |  |
| COPD | 1.17 |  |
| Bronchiectasis | 1.28 |  |
| Stroke | 1.14 |  |
| Solid tumor | 1.12 |  |
| Immunosuppressives | 1.26 |  |
| Influenza vaccination | 1.41 |  |
| Pneumococcal vaccination | 1.40 |  |
| ALB | 1.27 |  |
| Note: VIF values were calculated to assess multicollinearity among covariates in the multivariable cause-specific Cox regression model. VIF values <2 were considered to indicate no substantial multicollinearity.  Abbreviations: ALB, albumin; BMI, body mass index; COPD, chronic obstructive pulmonary disease; VIF, variance inflation factor. | | |

**Table S3.** Sensitivity analyses for the association between frailty and LRTI risk

| Analysis strategy | aHR (95% CI) | *P* value |
| --- | --- | --- |
| Fine-Gray competing risk model ^*^ | 1.29 (1.12–1.49) | <0.001 |
| Excluding participants with incomplete baseline data | 1.29 (1.10–1.52) | 0.002 |
| Exclusion of early LRTI cases (within 90 days) | 1.38 (1.13–1.69) | 0.002 |
| Right-censoring follow-up at 540 days | 1.32 (1.12–1.55) | <0.001 |
| Note: ^*^ For the Fine-Gray competing risk model, the reported values represent the adjusted subdistribution hazard ratio (aSHR), and this model was adjusted for age, sex, body mass index, smoking status, secondhand smoke, drinking status, heart failure, diabetes, bronchiectasis, chronic obstructive pulmonary disease, stroke, solid tumor, immunosuppressives, influenza vaccination, pneumococcal vaccination, and serum albumin level.  Abbreviations: aHR, adjusted hazard ratio; CI, confidence interval; LRTI, lower respiratory tract infection. | | |

**References**

1. Huang DT, Yealy DM, Filbin MR, et al. Procalcitonin-Guided Use of Antibiotics for Lower Respiratory Tract Infection. N Engl J Med. **2018** Jul 19;379(3):236-249.

2. Cao B, Huang Y, She DY, et al. Diagnosis and treatment of community-acquired pneumonia in adults: 2016 clinical practice guidelines by the Chinese Thoracic Society, Chinese Medical Association. Clin Respir J. **2018** Apr;12(4):1320-1360.

3. Metlay JP, Waterer GW, Long AC, et al. Diagnosis and Treatment of Adults with Community-acquired Pneumonia. An Official Clinical Practice Guideline of the American Thoracic Society and Infectious Diseases Society of America. Am J Respir Crit Care Med. **2019** Oct 1;200(7):e45-e67.

4. Global Initiative for Chronic Obstructive Lung Disease (GOLD). Global Strategy for Prevention, Diagnosis and Management of COPD: 2026 Report. 2026.

5. Woodhead M, Blasi F, Ewig S, et al. Guidelines for the management of adult lower respiratory tract infections--summary. Clin Microbiol Infect. **2011** Nov;17 Suppl 6:1-24.

6. Hill AT, Sullivan AL, Chalmers JD, et al. British Thoracic Society Guideline for bronchiectasis in adults. Thorax. **2019** Jan;74(Suppl 1):1-69.
